# Analytical model for effects of capsule shape on the healing efficiency in self-healing materials

**DOI:** 10.1371/journal.pone.0187299

**Published:** 2017-11-02

**Authors:** Zhong Lv, Songpeng Li, Huisu Chen

**Affiliations:** 1 School of Civil Engineering and Architecture, Anhui University of Technology, Ma’anshan, Anhui, the People’s Republic of China; 2 Jiangsu Key Laboratory of Construction Materials, School of Materials Science and Engineering, Southeast University, Nanjing, Jiangsu, the People’s Republic of China; Institute of Materials Science, GERMANY

## Abstract

The fundamental requirement for the autonomous capsule-based self-healing process to work is that cracks need to reach the capsules and break them such that the healing agent can be released. Ignoring all other aspects, the amount of healing agents released into the crack is essential to obtain a good healing. Meanwhile, from the perspective of the capsule shapes, spherical or elongated capsules (hollow tubes/fibres) are the main morphologies used in capsule-based self-healing materials. The focus of this contribution is the description of the effects of capsule shape on the efficiency of healing agent released in capsule-based self-healing material within the framework of the theory of geometrical probability and integral geometry. Analytical models are developed to characterize the amount of healing agent released per crack area from capsules for an arbitrary crack intersecting with capsules of various shapes in a virtual capsule-based self-healing material. The average crack opening distance is chosen to be a key parameter in defining the healing potential of individual cracks in the models. Furthermore, the accuracy of the developed models was verified by comparison to the data from a published numerical simulation study.

## Introduction

The initiation and propagation of damages/cracks at different length scales frequently result in structural degradation in materials. In structural materials, even micro-damage can lead to degradation in stiffness and durability and sometimes also to spontaneous loss of structural integrity. Furthermore, internal micro-damage is difficult to detect, repair cost of the damaged structural part is usually large and in some cases repair is impossible due to inaccessibility (for example, in space applications). In the meantime, the demand for continual improvement of engineering material performance is a common feature of many modern engineering projects and autonomous reliable repair of the internal micro-damage/cracks is desirable. To extend the structural lifetime and save maintenance costs, a fantastic strategy that once a crack or damage occurs the designed material possesses the ability to heal (recover/repair) the internal damages automatically by initiating some form of repair mechanism without any external intervention was put forward, i.e. self-healing [1, 2]. Generally, self-healing can be divided into autonomous and non-autonomous healing [2, 3]. Autonomic self-healing of cracks via capsules embedded which can prevent micro-crack growth into catastrophic macro-cracks in engineering materials is helpful to retain the reliability of structure, load-bearing capacity, and further the service life of structure [3]. A great achievements have been made in the self-healing materials with encapsulated healing agent, such as polymer composites[4, 5], cementitious materials [6–12], coatings [13, 14].

Self-healing of fracture surfaces in polymer composites with an encapsulated healing agent has rapidly developed over the past decade [5, 15–19]. A group from the University of Illinois at Urbana-Champaign first reported autonomous self-healing material by incorporating a microencapsulated healing agent and a catalyst chemical trigger in an epoxy matrix [20]. As the healing agent released by the microcapsules via capillary action happen to meet with the crushed catalyst particles, self-healing process will occur and the crack can be healed or sealed. From the perspective of the capsule shapes, spherical and elongated capsules (hollow tubes/fibres) are the main morphology of capsules used in the capsule-based self-healing materials [21, 22]. A key advantage of the spherical microencapsulation self-healing approach is the ease with which they can be incorporated within a bulk matrix and not significantly affect the performance of the material [13].The disadvantages are the need for microcapsule fracture and the need for the resin to encounter the catalyst prior to any repair occurring. Moreover, a undesirable drawback of spherical capsules in the self-healing system is that they do not allow long distance transport of the healing agent towards the crack and sufficiently supply volumes of healing agent to the damaged site [19]. In order to enhance the release of healing agent per crack area, Mookhoek et al. [23, 24] stated the necessary of the introduction of elongated liquid filled capsules and concluded that the self-healing efficiency could be improved by elongated capsules comparison with spherical capsules in capsule-based self-healing systems. Comparing with self-healing materials using spherical capsules, the strategy of elongated capsules (including hollow fibers with large aspect ratio like) employed has two advantages: one is that elongated capsules contain more agent than spherical microcapsules; the other is that elongated capsules with a weaving network structure can greatly enhance the mechanical properties of the matrix materials.

Much progresses that a crack hits the embedded spherical/elongated capsules has been made by modeling methods and numerical simulation [23, 25–28]. The fundamental requirement for the self-healing process to occur is that cracks need to approach/penetrate the capsules and cause to be ruptured and the healing agents release to fractured plane. Once the structure made up by self-healing materials has been subjected to damage it is extremely crucial to know what the chance that the structure can be healed is or how the healing efficiency of healing agent released of embedded capsules is. Evidently, only if the capsules are ruptured and the healing agent are released, the healing process may happen and the efficiency of self-healing will come. In other words, the more the embedded capsules are ruptured, the higher the potential efficiency of self-healing will grow. But it is unrealistic that all the embedded capsules can be crashed by cracks. Hence, the probability of a crack intersecting capsules in capsule-based self-healing system is brought forth. When the probability grows, both the probability of the capsules to be ruptured and the efficiency of healing agent released from capsules will accordingly increase. Hence, investigating the probability of a crack intersecting capsules will be helpful to provide critical insight in the selection of optimal self-healing material system [26] in addition to the conventional self-healing chemistry and mechanical characterization studies. Recently, Lv et al. [27, 29–31] applied geometrical probability theory to obtain the exact amount of capsules required to completely or partially repair the cracks in two- and three-dimensional capsule-based self-healing materials for certain specific scenarios. With the help of elementary probability principles, Zemskov [25] has developed analytical self-healing models to compute the probability of crack intersecting an encapsulated particle in the two-dimensional capsule-based self-healing cementitious model materials. These developed models gave some suggestions to fix critical crack lengths, ideal capsule size, and mean inter-capsule distance and facilitated to investigate the efficiency of healing agent of capsules in a self-healing material. Based on the probability of a crack hitting spherical capsules with a certain diameter calculated by Monte Carlo simulations, Huang [32] attempted to investigate the effects of the dosage and the size of capsules on self-healing efficiency in capsule-based self-healing material. From the view of capsule shape, many researches on the healing efficiency of capsules focused on the spherical capsules. Therefore, when spherical/elongated capsules are randomly distributed in the matrix and a crack occurs and grows, quantitative characterization of effect of capsule shape on the self-healing efficiency from viewpoint of probability will put forward the prospect of future research of the self-healing efficiency of capsules.

Ignoring all other aspects, the amount of healing agents released into the crack is essential to obtain a good healing. To enhance the release of healing agent per crack area, the introduction of elongated liquid filled capsules was recommended [23]. By an elementary probabilistic knowledge and numerical simulation, Mookhoek [23] performed a numerical study into the effect of aspect ratio, volume fraction and orientation of elongated capsules on the healing of liquid healing agent based systems. The probability of crack meeting for spherical capsules was developed when capsules are distributed at random in the matrix. However, the analytical express of probability of crack meeting for elongated capsules is an opening. Hence, a more comprehensive work on the capsule shape on the efficiency of healing agent released is essential.

The focus of this contribution is the description of the effects of capsule shape on the healing efficiency of healing agent released to crack surface in capsule-based self-healing materials within the framework of the theory of geometrical probability and integral geometry. Employing these tools, analytical expressions are developed to characterize the healing efficiency attributed for various shapes of capsules. The model stresses the crack opening distance, rather than the crack length, as a key parameter to characterize the healing potential of individual cracks. Furthermore, the accuracy of the developed analytical model was verified by comparison of its results with those of the published numerical simulation studies.

## Theoretical model

**Fig 1:**
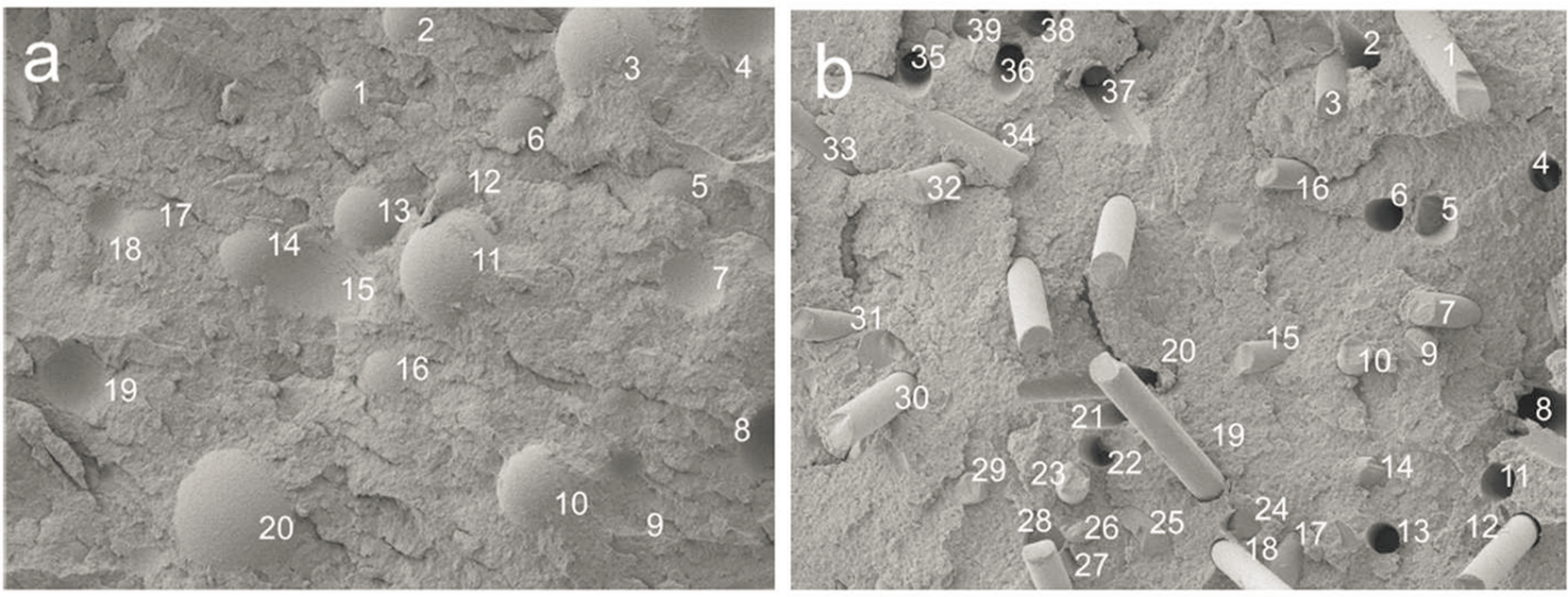
Experimental investigation (SEM) of the effect of capsule shape on healing efficiency in self-healing composite material [33], (a) a fracture surface of dispersed spherical capsules, (b) a fracture surface of dispersed cylindrical capsules.

The fracture surface of epoxy resin containing randomly dispersed spherical polymer particles or chopped glass fibres was experimentally examined to predict the self-healing efficiency of capsules by scanning electron microscopy (SEM) [33]. In the experiment, polymethylmethacrylate (PMMA) solid bodies spheres and short, high aspect ratio, glass fibres of similar volume were used. Fig 1 shows in self-healing composite material (a) a fracture surface of dispersed spherical capsules and (b) a fracture surface of dispersed cylindrical capsules.

Usually, the typical concentration of capsules mixed in self-healing material matrix is relatively low, such as in polymer composites [34, 35]. In other words, it can be considered that for a low concentration the embedded capsules will roughly present in a state of three-dimensional randomness from a viewpoint of spatial distribution. Moreover, it can be assumed that capsules act independently and are not clustered. Hence, the embedded capsules can be considered to have a uniform spatial distribution in the matrix. In the following sections of this paper we develop analytical models to characterize the effect of capsule shape on the healing efficiency for an arbitrary crack in a virtual capsule-based self-healing material matrix with randomly distributed embedded capsules. In this research, it is assumed that the planar cracks randomly appear and then directionally propagate in a three-dimensional matrix setup. Of course, not all capsules are hit by cracks and a probability value of a crack interaction with capsules will result. Furthermore, the thickness of the capsule wall is also ignored and the volume of the healing agent to be released by a capsule is therefore equal to the capsule volume.

### Analytical model of efficiency of healing agent released from spherical capsules

**Fig 2:**
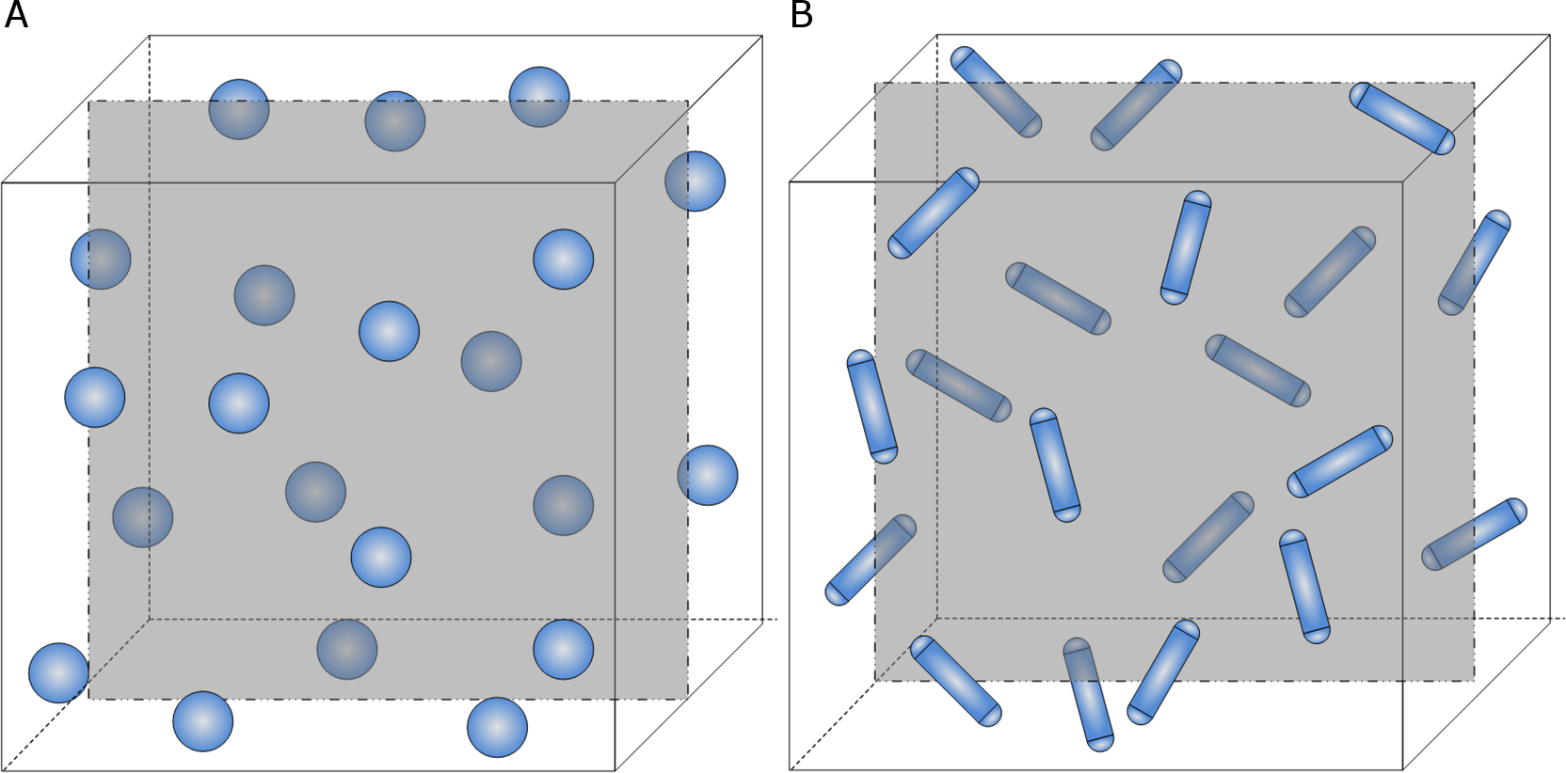
Schematic representation of the self-healing material matrix with a random sectioning planar crack, (a) spherical capsules, (b) spherocylindrical capsules.

As shown in Fig 2A, consider a countable number of congruent spherical capsules with radius *r* uniformly distributed in the cubic sample T of side *a* (i.e. a representative volume element (RVE) satisfying *r*<<*a*) and *V*_*V*_ denotes the volume fraction of capsules inside the sample. The capsule-based self-healing virtual material is considered to be a homogeneous matrix.

As it is deduced in the Appendix A, the probability that a spherical capsule in the cubic sample hit by a random sectioning planar crack is given by Eq (A.5). Then, the number of capsules, *n*, hit by a random sectioning plane with area *A*_*T*_ in the sample is

$$n=p\cdot N=p\cdot \frac{V_{V}\cdot V_{RVE}}{V_{0}}$$ (1)

in which *V*_0_ is the single capsule volume and *N* is the total amount of spherical capsules present inside the sampling region. So, the volume of healing agent released on the per area of crack is given by

$$X=\frac{n\cdot V_{0}}{A_{T}}$$ (2)

Alternatively, when a cubic sample is cut by a random section plane, the average cross-sectional area has been obtained by Eq (A.12). From Eqs (1) and (2), and *V*_*RVE*_ = *a*^3^, it obtains

$$X=2r\cdot V_{V}$$ (3)

In practice, it is unlikely for a ruptured capsule to fully release its content. The volume of healing agent to be released may be discounted because of the effect of the size and the shell materials of capsule and the viscosity of healing agent, etc. Consequently, the dosage of healing agent to be released should be discounted. Hence, a factor *f* (*f*<1) which characterizes the discount is necessary and well-defined. Namely, when a capsule is fractured upon intersection by a crack, a part of the healing agent with volume fraction *fV*_0_ is released and flows in the crack. So, repeating the above process the volume of healing agent released on the per area of crack becomes

$$X=\frac{n\cdot V_{0}\cdot f}{A_{T}}=2r\cdot f\cdot V_{V}$$ (4)

### Analytical model of efficiency of healing agent released from spherocylindrical capsule

We here call a cylindrical shape terminated by a half sphere at either end to be a spherocylinder, as illustrated in Fig 2B. Consider a countable number of congruent spherocylindrical capsules with height *h* and radius *r* uniformly distributed in the cubic sample T of side *a* (i.e. a representative volume element (RVE) satisfying *h*, *r* << *a*) and *V*_*V*_ denotes the volume fraction of capsules inside the sample. The capsule-based self-healing virtual material is considered to be a homogeneous matrix. As it is deduced in the Appendix A, the probability that a spherocylinder in the cubic sample hit by a random sectioning planar crack can be expressed by Eq (A.10). Then, the number of spherocylindrical capsules, *n*, hit by a random sectioning plane in the sample is

$$n=p\cdot N=p\cdot \frac{V_{V}\cdot V_{RVE}}{V_{0}}$$ (5)

in which *V*_0_ is the single capsule volume and *N* is the total amount of spherocylindrical capsules being present inside the sampling region. So, the volume of healing agent released on the per area of crack is given by

$$Y=\frac{n\cdot V_{0}}{A_{T}}$$ (6)

Alternatively, when a cubic sample is cut by a random section plane, the average cross-sectional area has been obtained by Eq (A.12). From Eqs (5) and (6) and *V*_*RVE*_ = *a*^3^, it obtains

$$Y=\frac{h+4r}{2}\cdot V_{V}$$ (7)

Denote *τ* the aspect ratio of a spherocylindrical capsule, that is, *h* = 2*r*(*τ*-1). Then Eq (7) becomes

$$Y=\left(\tau +1\right)r\cdot V_{V}$$ (8)

For the case of spherocylindrical capsule, a similar discussion can be conducted when only a fraction of the capsule content is released from the embedded capsules as was done for the spherical capsules. Namely, a factor *f* (*f* <1) which characterizes the discount is interesting.

## Results and discussion

### Verification for spherical capsule

When spherical capsules are employed in the self-healing system, the analytical expression Eq (3) of self-healing efficiency of capsules is identical with Eq (5) presented elsewhere [23]. It is clearly shown that with increasing capsule size the total amount of healing agent released increases rapidly. From the development procedure of the analytical expression it can be seen that Eq (3) is on basis of a mathematically rigorous reasoning and is more comprehensive. The article stated ‘a test plane at a random position but parallel to one of the faces of the RVE axes was defined’ [23], while in this contribution a random sectioning planar crack cutting the sample is sufficient.

**Fig 3:**
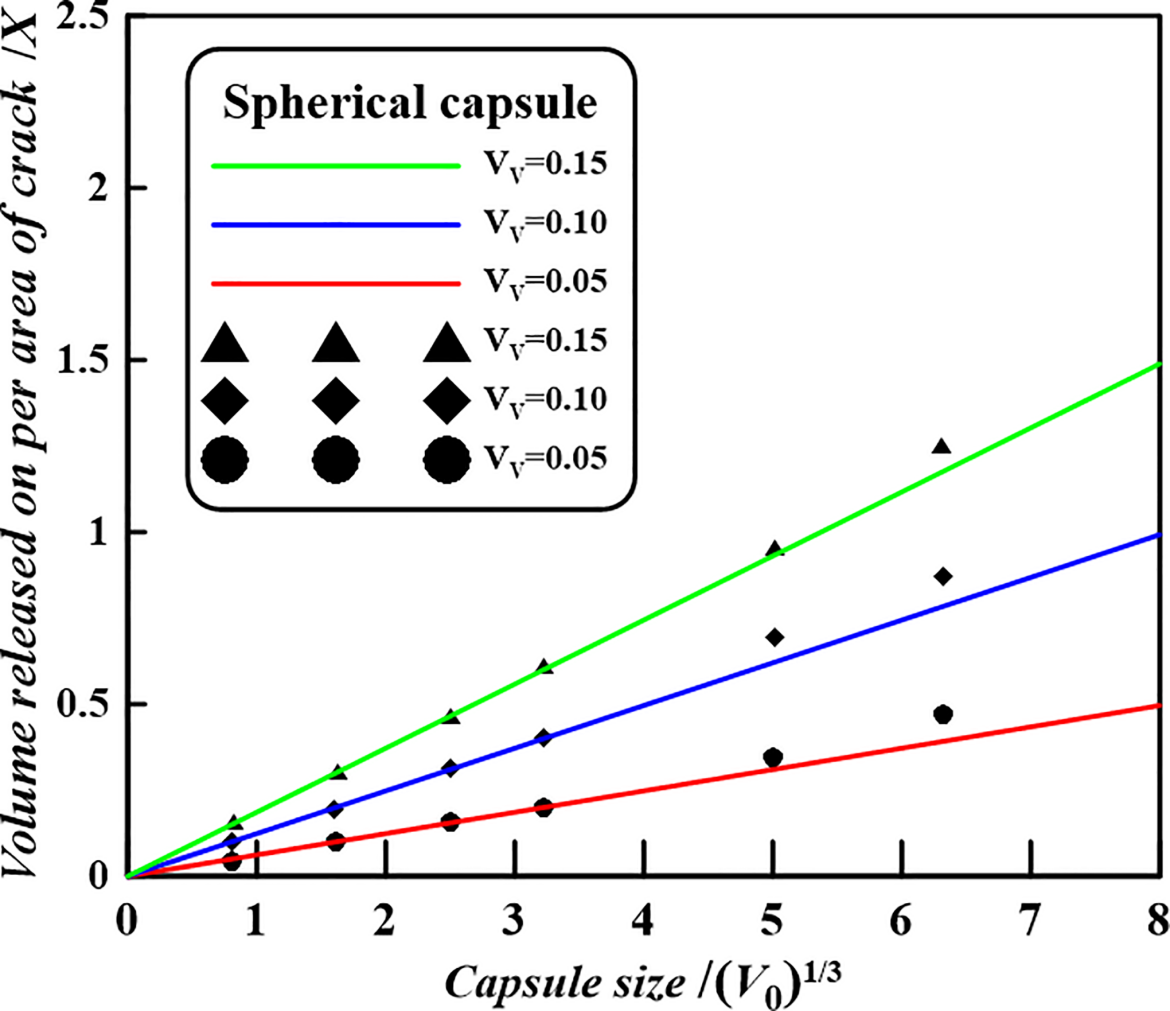
Theoretical volume of healing agent released per area as a function of the spherical capsule size for three volume fractions.

In the case of spherical capsules with capsular volume of *V*_0_ = 15.7 μm^3^ (i.e. a capsule diameter 2*r* = 3.11 μm) and volume fraction *V*_*V*_ = 0.10, an average volume per area is found of *X* = 0.3107 μm^3^/μm^2^ via the presented formula Eq (3). This value is consistent with the numerical result given in [23]. Therefore, the volume released would be capable of filling a crack volume with a uniform crack opening distance of maximum 0.3107 μm^3^/μm^2^. Fig 3 shows the calculated average volume per area as a function of the capsule radius for three volume fractions. The observed linear dependence between average volume released per crack area and capsule size (*V*_0_)^1/3^, for a given volume fraction *V*_*V*_, can simply be derived from Eq (3). That is,

$$X=2\cdot {\left(V_{0}\right)}^{1/3}\cdot {\left(\frac{3}{4\pi }\right)}^{1/3}\cdot V_{V}$$ (9)

As shown in Fig 3, for given volume fraction *V*_*V*_ = 0.05, 0.1, 0.15 and capsule size (*V*_0_)^1/3^ = 0.81, 1.61, 2.51, 3.22, 5.01, 6.31 μm, it can be drawn the line graph of *X* via (*V*_0_)^1/3^ according to Eq (9). Meanwhile, for given *V*_*V*_ and *V*_0_ the specific numerical values of the volume of healing agent released on the per area of crack were numerically investigated (S1 Excel) [23]. It is found that the simulation results of the self-healing efficiency *X* of the volume of healing agent released on the per area of crack vs. capsule size (*V*_0_)^1/3^ is consistent with the trend as demonstrated in Eq (9). Hence, the presented analytical model could be verified. By the way, the scattered points which stand for the simulation values in the Fig 3 were extracted from Fig 2B in the paper [23].

### Verification for spherocylindrical capsule

**Fig 4:**
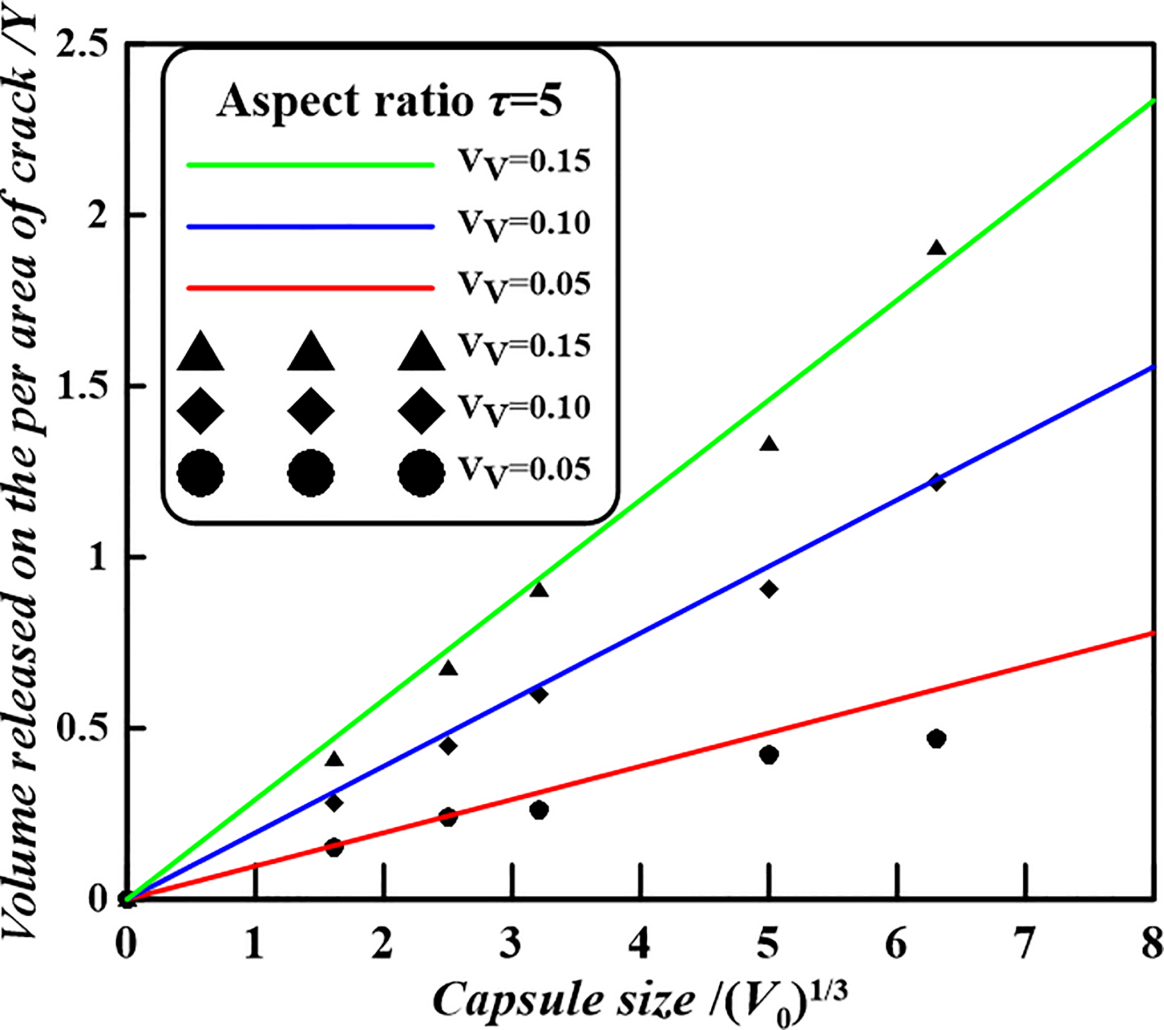
Theoretical volume of healing agent released per area as a function of the spherocylindrical capsule size for given aspect ratio.

In the case of spherocylindrical capsules with a capsular volume of *V*_0_ = 15.7 μm^3^ and an aspect ratio *τ* = 5 (i.e. *h* = 8*r*) and volume fraction 0.10, an average volume per area is found of *Y* = 0.487 μm^3^/μm^2^ via formula Eq (8). While for the identical capsular volume, aspect ratio and volume fraction of spherocylindrical capsule, a numerical value, 0.448 μm^3^/μm^2^, of the volume of healing agent released on the per area of crack was given in Ref. [23]. It can be found that the numerical result nearly approaches to the presented analytical value. The volume released would be capable of filling a crack volume with a uniform crack opening distance of maximum 0.487 μm^3^/μm^2^. Fig 4 shows the theoretical average volume per area as a function of the capsule size for three volume fractions. The observed linear dependence between average volume released per crack area and the size of a spherocylindrical capsule (*V*_0_)^1/3^, for a given volume fraction *V*_*V*_, can easily be derived by Eq (8). As shown in Fig 4, for given volume fraction *V*_*V*_ = 0.05, 0.1 and 0.15, aspect ratio *τ* = 5 and spherocylindrical capsule size (*V*_0_)^1/3^ = 1.69, 2.49, 3.21, 5.00, 6.31 μm, graph of *Y* with respect to (*V*_0_)^1/3^ on the basis of Eq (8) can be drawn. It should be noted that there is a simply relation

$$Y=\frac{\left(\tau +1\right)\cdot V_{0}^{1/3}\cdot V_{V}}{{\left(2\pi \tau -\frac{2}{3}\pi \right)}^{1/3}}$$ (10)

At the same time, for given *V*_*V*_ and *V*_0_ the specific numerical results (i.e. the scattered points in the Fig 4) of the volume of healing agent released on the per area of crack are borrowed from the published paper (S2 Excel) [23]. It is found that from Fig 4 the simulation results of the self-healing efficiency *Y* of the volume of healing agent released on the per area of crack vs. capsule size (*V*_0_)^1/3^ is consistent with the trend as demonstrated in Eq (8). Hence, the presented analytical results could be verified.

**Fig 5:**
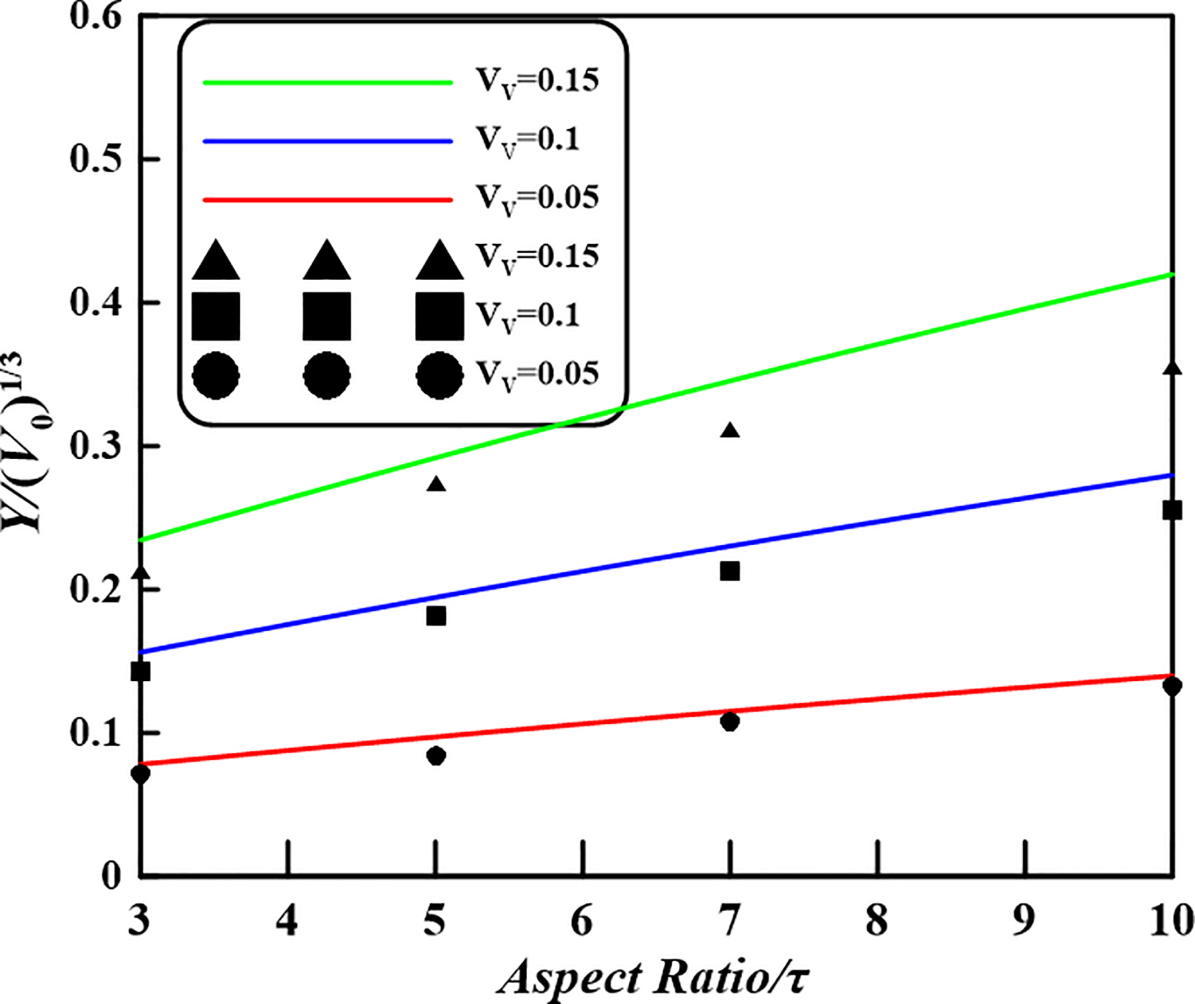
Comparison the analytical curves of the self-healing efficiencey characterized by Y/ (*V*_0_)^1/3^ with the numerical values.

Also, the relationship between the efficiency and the aspect ratio can be investigated from Eq (10). Here, *Y*/ (*V*_0_)^1/3^ for different capsule concentrations and aspect ratio at random capsule orientation can be obtained. From the analytical expression *Y*, i.e. Eqs (8) and (10), it can be concluded that *Y*/ (*V*_0_)^1/3^ is a function of the aspect ratio *τ* and volume fraction *V*_*V*_. Both the curve *Y*/ (*V*_0_)^1/3^ and the numerical values (S3 Excel) given by [23] are illustrated in Fig 5. It appears that for a given volume fraction there is a linear dependence between *Y*/ (*V*_0_)^1/3^ and *τ*. From Fig 5 the self-healing efficiencey characterized by *Y*/ (*V*_0_)^1/3^ is improved increases as the aspect ratio increases for a given healing capacity of a single capsule. When the volume fraction and aspect ratio of embedded sphereocylindical capsules gets bigger, the deviations bewteen theoretical results and simulated values of *Y*/ (*V*_0_)^1/3^ also grows. The deviations may be originated from the ignoring of the overlapping among the embedded capsules, especially as the aspect ratio and the content are both increasing. Furthermore, for real materials containing elongated particles at a higher volume fraction (e.g. *V*_*V*_>0.1) alignment of the particles is almost unavoidable and isotropic distributions are unrealistic.

### The effect of capsule shape on the efficiency of healing agent to be released

We now consider the influences of capsule shape on the efficiency of healing agent to be released. If the volume fraction of capsules embedded is fixed both for spherical and spherocylindrical capsules, *Y*/*X* which represents the ratio of the volume of healing agent to be released on the per area of crack can be obtained from Eqs (9) and (10) as follows

YX=τ+12⋅(2σ3τ−1)1/3(τ>1,withσ=V0-cylV0−sph) (11)

**Fig 6:**
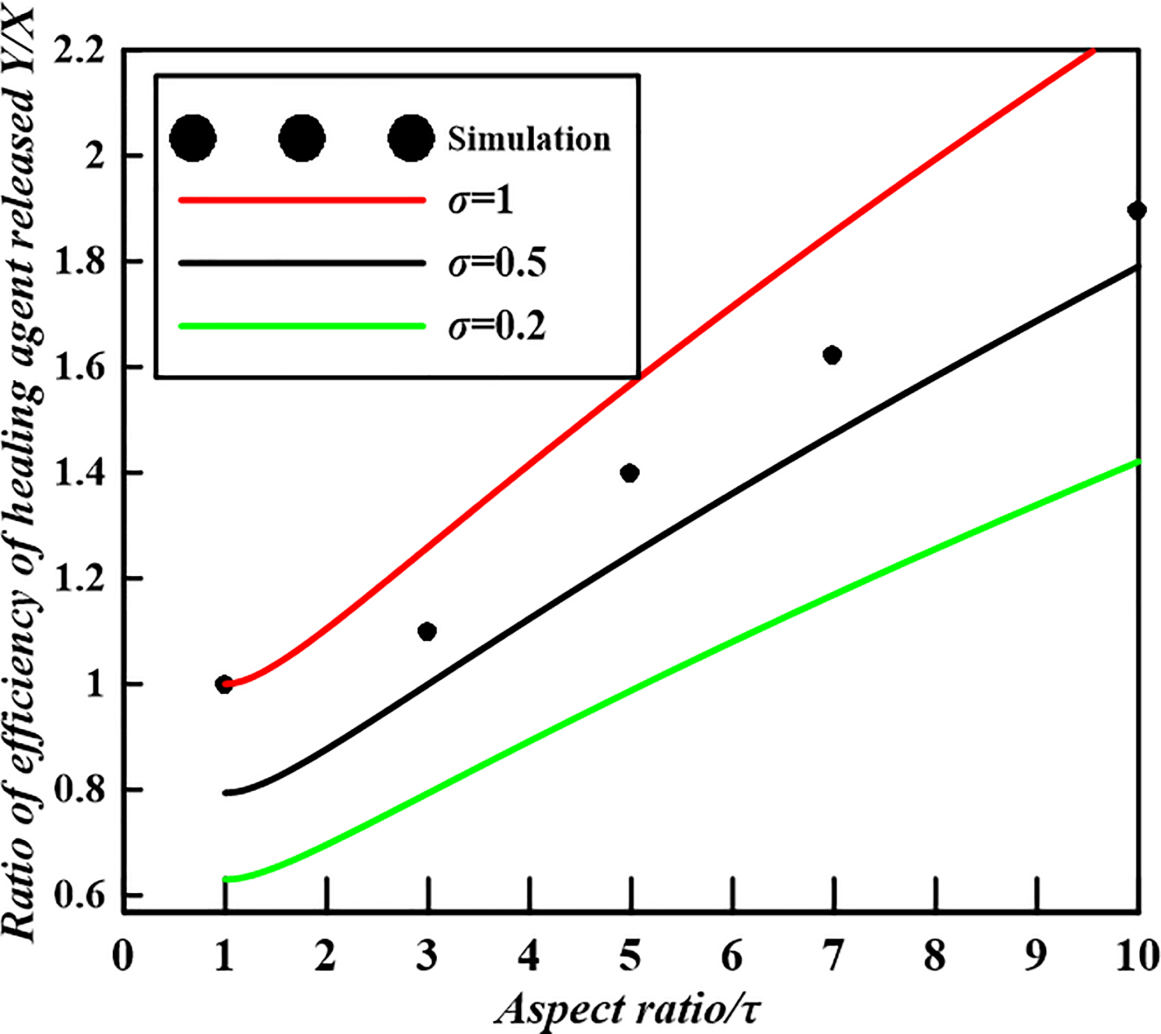
The effect of capsule shape on the efficiency of healing agent released with a given ratio of capsule volume for spherical and spherocylindrical capsules.

Actually, *Y*/*X* is an analytical formula that characterizes the quotient of the determined slopes for spherocylindrical and spherical capsules at a fixed total capsule volume fraction. If the volume fraction of two types of capsules are equal and the size of spherical capsules with fixed volume which will be embedded in the matrix is given, the efficiency of healing agent released from spherocylindrical capsules employed in self-healing system increases as the aspect ratio or the volume of the type of spherocylindrical capsule grows as shown in Fig 6. The theoretical results taking *σ* = 0.2, 0.5, 1 (red solid line in Fig 6) as examples are obtained from the analytical model, i.e. Eq (11), while the numerical values (S4 Excel) are given only for σ = 1 in the ref. [23]. Specifically, only when *σ*≥1, *Y*/*X* is larger than 1. That is to say, the efficiency of any elongated capsules is higher than that of spherical ones. For *σ*<1, the *Y*/*X* is not always more than 1. At this situation, *Y*/*X* is not only related with the aspect ratio, but it also involves the size of capsules (i.e. capsule volume) embedded. Just when *σ* = 1 and *τ* = 1, the efficiencies are equal for the two types of capsules. Therefore, both shape and size of capsules should be jointly investigated to improve the efficiency of healing agent released in capsule-based self-healing composite materials. This point is opposed with the claim [23] that ‘for randomly positioned cylindrical microcapsules the *RIF* is a function of the capsule aspect ratio only and is independent of the volume fraction and the capsule volume’.

## Conclusions

In this contribution the effects of capsule shape on the efficiency of healing agent to be released in capsule-based self-healing composite materials are conducted. When capsules are randomly distributed in the matrix and a crack occurs and grows, the analytical expression of efficiency of healing agent released by spherical or spherocylindrical capsules are stemmed from a mathematically rigorous reasoning via the theory of geometrical probability and integral geometry. The crack opening distance is chosen to characterize the healing potential of individual cracks in the models. Furthermore, the reliabilities of the developed models can be verified by data from relevant literature information, which was implemented via numerical simulation. It was found that both shape and size of capsules should be jointly investigated to improve the efficiency of healing agent to be released in capsule-based self-healing composite materials. This point is opposed with the existing claim. As a result of the model, the volume fraction of capsules required to be embedded in matrix can be determined via the developed efficiency model for different type of shaped capsules. The results of the analytical models will serve to understand the probability of crack intersection with capsules and guide the further development of spherical or elongated liquid filled capsules in capsule-based self-healing composite materials.

## Appendix A

The probability that a randomly chosen crack plane meets the capsule could be obtained by the theory of Integral Geometry as follows.

Let *K*_0_, *K*_1_ be convex sets in *E*_*n*_ such that *K*_1_ ⊂ *K*_0_. The probability that a randomly chosen *r*-plane *L*_*r*_ (*r* = 1, 2, …, *n*-1) that meets *K*_0_ also meets *K*_1_ is

$$p\left(L_{r}\cap K_{1}\neq \emptyset \right)=\frac{W_{r}(K_{1})}{W_{r}(K_{0})}=\frac{M_{r-1}(\partial K_{1})}{M_{r-1}(\partial K_{0})}$$ (A.1)

where, *M*_*r*_(*∂K*) = *nW*_***r+*1**_(*K*) (*r* = 0, 1, …, *n*-1), and *n* is the dimension of space. *W*_***r+*1**_(*K*) is often called *quermassintegrale* or mean cross-sectional measures of a convex set *K* introduced by Minkowski, while *M*_*r*_(*∂K*) is the integrals of mean curvature of the boundary *∂K* of a convex set *K* [36]. For *n* = 3, *r* = 2, it becomes

$$p\left(L_{2}\cap K_{1}\neq \emptyset \right)=\frac{W_{2}(K_{1})}{W_{2}(K_{0})}=\frac{M_{1}(\partial K_{1})}{M_{1}(\partial K_{0})}$$ (A.2)

In the capsule-based self-healing model material it equates a convex body *K*_1_ with a capsule, a *K*_0_ with a sampling region (or RVE, a cube). For a spherical capsule *K*_1_ with radius *r*, it holds [36]

$$W_{2}\left(K_{1}\right)=\left(4\pi r\right)/3,$$ (A.3)

For the cubic RVE *K*_0_ with edge *a*, it has

$$W_{2}\left(K_{0}\right)=\pi a,$$ (A.4)

Hence, from Eqs (A.3) and (A.4), when a spherical capsule is embedded in the sampling region, the probability that a randomly chosen crack plane that meets the sampling region *K*_0_ also meets the capsule *K*_1_ is

p=(4r)/(3a) (A.5)

For spherocylindrical capsule the concept of *Parallel convex sets* in the theory of Integral Geometry is employed. *Parallel convex sets*: The parallel body *K*_*ρ*_ in the distance *ρ* of a convex set *K* is the union of all solid spheres of radius *ρ* the centers of which are points of *K*. The boundary *∂K*_*ρ*_ is called the parallel surface of *∂K* in the distance *ρ*. It is worth noting that the integral of mean curvature of the boundary *∂K*_*ρ*_ can be expressed by the function of the integral of mean curvature of the boundary *∂K* [36],

$$M_{i}(\partial K_{\rho })=\sum_{j=0}^{n-i-1}\left(\begin{array}{c} n-i-1\\ j \end{array}\right)M_{i+j}(\partial K)\rho^{j},j=i=0,1,\ldots ,n-1.$$ (A.6)

So, a spherocylindrical capsule, i.e., a cylindrical shape terminated by a half sphere at either end, can be seen as a parallel body *K*_*r*_ in the distance *r* of a segment set *K* with length *h* is the union of all solid spheres of radius *r* the centers of which are points of *K*. From Eq (A.6) with *n* = 3, *i* = 1, it has

$$M_{1}\left(\partial K_{r}\right)=M_{1}\left(\partial K\right)+M_{2}\left(\partial K\right)r.$$ (A.7)

For the ordinary space *E*_3_, when *K* reduces to a line segment of length *h*, the mean curvature integrals satisfies *M*_1_(*∂K*) = *πh*, *M*_2_(*∂K*) = 4*π*. So, the mean curvature integrals of a spherocylindrical capsule is

$$M_{1}\left(\partial K_{r}\right)=\pi h+4\pi r$$ (A.8)

Namely,

$$W_{2}\left(K_{r}\right)=\left(\pi h+4\pi r\right)/3.$$ (A.9)

Hence, from Eqs (A.4) and (A.9), when a spherocylindrical capsule is embedded in the sampling region, the probability that a randomly chosen crack plane that meets the sampling region *K*_0_ also meets the capsule *K*_1_ is

p=(h+4r)/(3a) (A.10)

It is known that the average sectioning area of a convex sample cutting by a plane is [36]

$$A_{T}=\left(2\pi V_{T}\right)/M_{1}$$ (A.11)

where *M*_1_ is integral of mean curvature of the boundary and *V*_*T*_ is the volume of convex sample.

Therefore, from Eqs (A.4) and (A.11), the average area of a cube with edge *a* sectioned by a plane is

$$A_{T}=\left(2a^{2}\right)/3.$$ (A.12)

For a convex body *K* in three-dimensional space, the integrals of mean curvature (mean curvature integrals) are *M*_0_ = area of the boundary *K*; *M*_1_ = integral of mean curvature of the boundary *K*. Then, the integrals of mean curvature can be expressed as follows [36]

$$M_{1}=\frac{1}{2}\int_{U_{2}}\Delta du_{2}$$ (A.13)

where *Δ* is the breadth of *K* in the direction *u*_2_, that is, the distance between the two support planes of *K* that are perpendicular to *u*_2_ and that contain *K* between them. The breadth of *K* is also called as the mean caliper diameter in the terminology of stereology [37]. Thus we have the mean values

$$\Delta =M_{1}/2\pi .$$ (A.14)

It follows from the Eq (A.14) that *M*_1_ is equal, up to the factor 2*π*, to the mean distance between parallel support planes.

## Supporting information

S1 Excel Numerical simulation data borrowed from Ref. [23] for Fig 3. (XLS)

S2 Excel Numerical simulation data borrowed from Ref. [23] for Fig 4. (XLS)

S3 Excel Numerical simulation data borrowed from Ref. [23] for Fig 5. (XLS)

S4 Excel Numerical simulation data borrowed from Ref. [23] for Fig 6. (XLS)
